# Shear wave elastography in the diagnostics of parathyroid adenomas–new application of the method

**DOI:** 10.1007/s12020-018-1553-0

**Published:** 2018-02-21

**Authors:** Adam Stangierski, Kosma Wolinski, Marek Ruchala

**Affiliations:** Department of Endocrinology, Metabolism and Internal Medicine, Poznan University of Medical Sciences, Poznan, Poland

**Keywords:** Elastography, Shear wave elastography, Ultrasonography, Parathyroids, Parathyroid adenoma

## Abstract

**Purpose:**

Shear wave elastography (SWE) was described as valuable tool in the diagnostics of distinct types of thyroid lesions, thyroiditis and several other non-thyroidal conditions, such as liver inflammation and fibrosis or diagnostics of breast lesions. The aim of the current study was to assess the appearance of parathyroid adenomas in SWE and to check prospectively if SWE can be valuable additional tool in the diagnostics of pathologically enlarged parathyroids.

**Methods:**

Patients with parathyroid adenomas confirmed by histopathology were included. Subjects with benign thyroid lesions were enrolled to the control group. Elasticity of parathyroid adenomas and benign thyroid nodules was measured and compared.

**Results:**

Sixty five patients with parathyroid adenomas and 35 patients with 51 benign thyroid nodules were included. Parathyroid adenomas where significantly more elastic than benign thyroid nodules–mean elasticity of the lesion was 5.2 ± 7.2 vs. 24.3 ± 33.8 kPa, respectively. Relative mean elasticity (in comparison with surrounding thyroid tissue) was 0.30 ± 0.36 and 2.8 ± 3.9, respectively.

**Conclusions:**

SWE can be useful tool in the diagnostics of parathyroid adenomas. Enlarged parathyroids are significantly more elastic than benign thyroid lesions. Low elasticity of the lesion constitutes feature with high negative prognostic value, allowing for reliable exclusion of suspicion of parathyroid adenomas.

## Introduction

Elastography (ES) is a term encompassing methods of the tissue stiffness assessment [1–4]. There are numerous variants of the technique. Strain ES is older variant, which is operator-dependent, requires unaffected tissue for comparison with region of interest (e.g., focal lesion) and the interpretation is rather subjective [2]. Shear wave elastography (SWE) is novel and promising type of ES. It is believed to be more objective, reproducible and reliable as it does not require any compressive maneuvers, results are expressed as absolute values, comparison with other structures is not needed [2, 5, 6]. SWE was described as valuable tool in the diagnostics of distinct types of thyroid lesions such as thyroid cancer [5–8], chronic [9, 10] and subacute thyroiditis [9, 11] and other non-thyroidal conditions, such as liver inflammation and fibrosis [12], diagnostics of breast lesions or prostate cancer [13, 14].

The aim of the current study was to assess the appearance of parathyroid adenomas in SWE and to check prospectively if SWE can be valuable additional tool in the diagnostics of pathologically enlarged parathyroids.

## Materials and methods

### Patients

The Poznan University of Medical Sciences Ethical Committee approved this study and all participants provided informed written consent to participate in it. Patients with suspicion of primary hyperparathyroidism referred to the department of endocrinology or endocrine outpatient clinic between January 2012 and December 2015 were assessed. In order to avoid potential confusions and errors in the correlation of elastography and final histopathology of the assessed nodule, patients with multiple lesions in the thyroid lobe containing enlarged parathyroid were not included to the study. Patients who met the ATA criteria for surgical intervention [15] were included for the final analysis. After subsequent referral to the surgery, presence of parathyroid adenomas was confirmed by postoperative histopathology. Randomly selected patients with benign thyroid nodules admitted in the outpatients clinic were included to the control group. Thyroid nodules confirmed as benign (Bethesda II) in cytological evaluation [15] were finally included.

### Ultrasonography and SWE

As a first step conventional ultrasonography was performed using the AIXPLORER system with 2–10 MHz linear transducer (Supersonic Imagine, Aix enProvence, France). The standard procedure included the description of the diameter, shape, echogenicity, presence of calcifications and localization in each of the visualized lesions.

Second step, shear wave elastography, was performed just after conventional ultrasonography. Elasticity of each lesion was assessed quantitatively and expressed in kPa. Data on the minimal, mean and maximal elasticity of the lesions and adjacent thyroid tissue were recorded. The measurements were obtained only from the solid, non-calcified parts of each lesion. All examinations were assessed by the co-operating endocrinologists with 7 and more than 20 years of experience in ultrasonography of thyroid disorders.

### Statistical analysis

All calculations were performed using Statistica v.12 software with medical package (from StatSoft); p-value under 0.05 was considered significant.

## Results

**Fig. 1:**
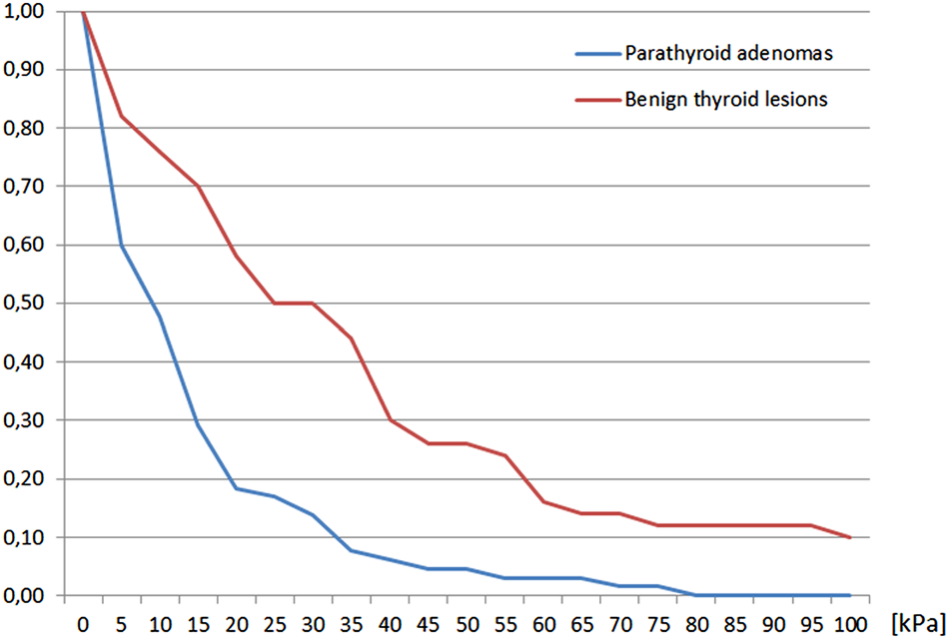
Proportion of parathyroid adenomas and benign thyroid lesions exceeding given Q-box max values

**Fig. 2:**
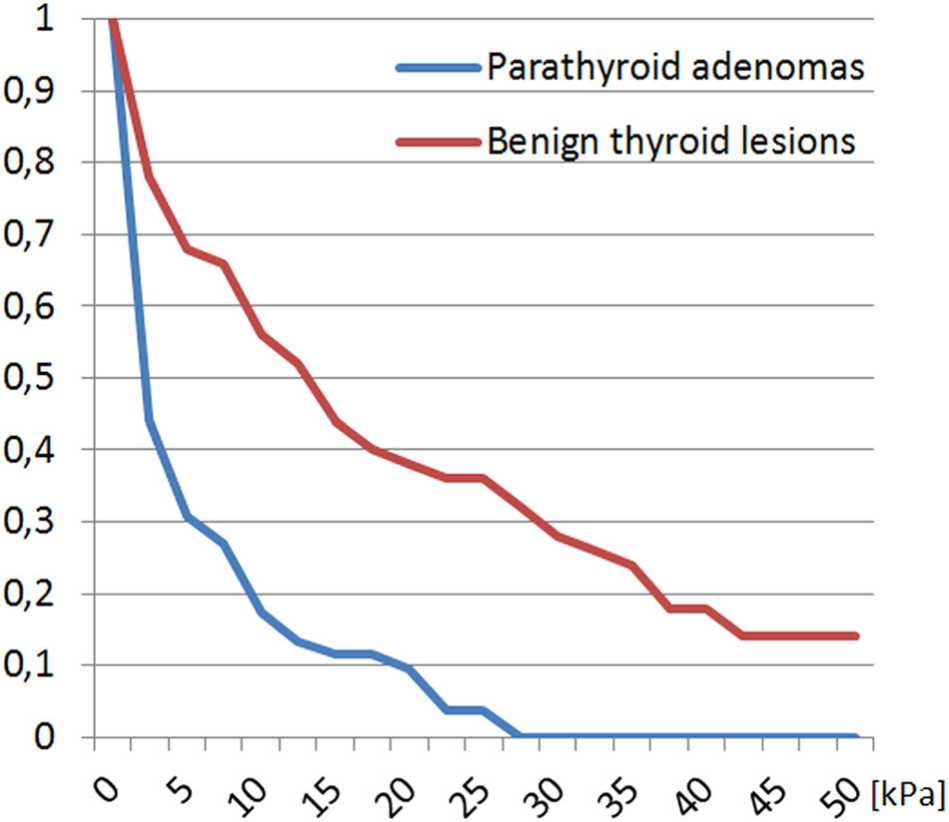
Proportion of parathyroid denomas and benign thyroid lesions exceeding given Q-box mean values

**Fig. 3:**
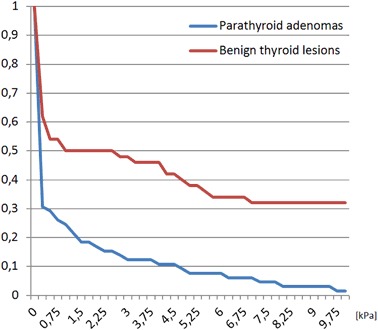
Proportion of parathyroid denomas and benign thyroid lesions exceeding given Q-box min values

**Fig. 4:**
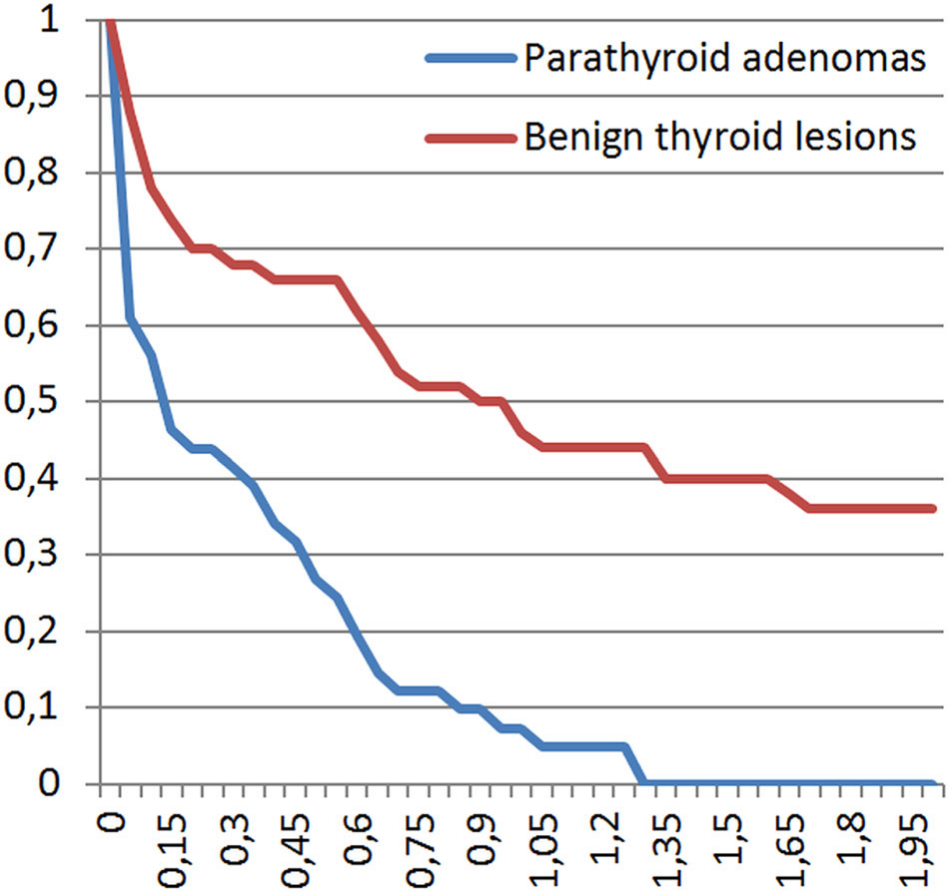
Relative Q-box mean for parathyroid adenomas and benign thyroid lesions calculated as elasticity of the lesion divided by elasticity of neighboring thyroid tissue

**Fig. 5:**
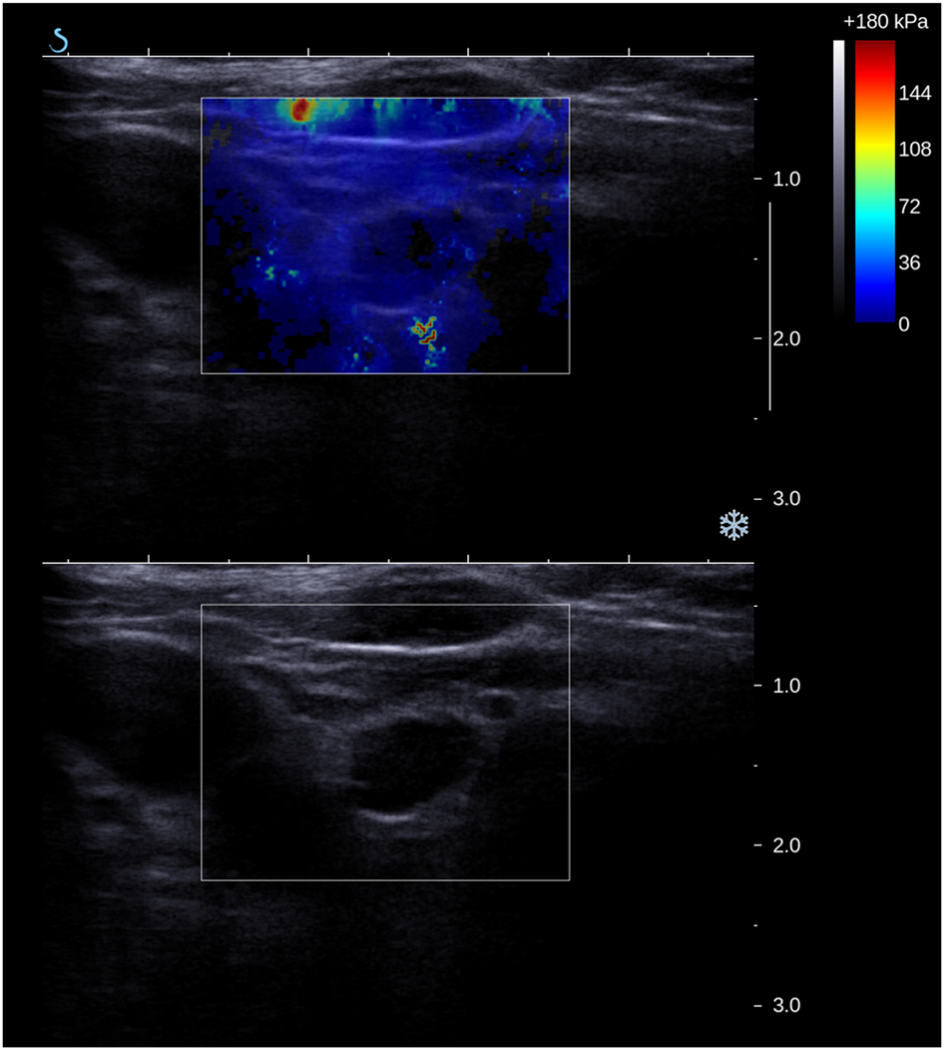
Example of parathyroid adenoma appearance in Shear wave elastography and conventional ultrasonography

Sixty five patients with parathyroid adenomas were included. There were 60 women and five men, mean age was 57.6 years with standard deviation (SD) equal to 13.8 years. The average size of the lesion (maximal diameter) was 14.9 mm with SD equal to 7.7 mm. Thirty five patients with 51 benign thyroid nodules were enrolled as a control group. There were 30 women and five men, mean age was 58.9 years with SD equal to 16.0 years. The average size of the lesion (maximal diameter) was 11.8 mm with SD equal to 6.5 mm. The differences regarding age-distribution and sex-distribution were statistically not significant (*p* > 0.05), the difference in average lesion size between the groups was of borderline significance (*p* = 0.03). Comparison of mean, minimal and maximal elasticities of parathyroid adenomas and thyroid nodules is given in Table 1 and Figs. 1 and 2. Relative elasticities of lesions (in comparison to surrounding thyroid tissue) are given in Table 2 and Figs. 3 and 4. Example of appearance of PA in SWE and conventional US was given on Fig. 5.

## Discussion

Primary hyperparathyroidism (PH) is a medical condition usually caused by parathyroid adenoma (PA) [15]. The prevalence depends on population and was reported to be from 76 to 233 per 100,000 women and from 30 to 85 per 100,000 men [16].

Untreated PH can lead to several complications, such as osteoporosis, nephrolithiasis, gastrointestinal ulcerations or psychiatric disorders [17]. Whereas normal parathyroids are not visible in US thyroid examination, PAs usually can be visualized as hypoechogenic lesions localized near the thyroid capsule, in the rear part of the gland [15]. Presence of such lesion should always arise suspicion of pathologically enlarged parathyroids. However, PAs are occurring distinctly less commonly than nodules deriving from thyroid tissue. Consequently, in the vast majority of cases such lesions are just thyroid lesions and the biochemical and scintigraphic diagnostics is somehow unnecessary. This fact indicates strong need for additional diagnostic tools for preliminary differentiation between thyroid and parathyroid lesions.

SWE is a novel sonographic (US) technique which allows for the assessment of tissue elasticity. Numerous studies assessing usefulness of SWE in the differentiation of benign and malignant thyroid lesions have been published [5, 18, 19]. Most of them indicated high diagnostic value of the technique [6, 20]. However, there is very limited amount of studies concerning use of SWE in diagnostics of parathyroid adenomas – particular group of lesions which can be visualized during neck ultrasonography.

We aimed to check the utility of all available elastographic parameters–absolute and relative minimal, mean and maximal elasticities of the parathyroid lesions to obtain information which of the parameters should be considered as most important in the diagnostics of parathyroid lesions. Maximal and minimal elasticity of the lesions (Q-box max and Q-box min respectively) refers to the most and the least elastic pixel of the region of interest (ROI), whereas whole ROI is included in the calculation of mean elasticity (Qbox mean) what makes this parameter less susceptible to artifacts and outliers outcomes. We also aimed to check the relative elasticity of PAs and thyroid lesions–in comparison to thyroid parenchyma.

According to our results there are significant differences between enlarged parathyroids and benign thyroid lesions in SWE examination. Parathyroids were significantly more elastic–Qbox mean was almost five times lower than in case of BTL (benign thyroid lesions), median–over six times lower. Significant differences concerned other measured paramethers–Q-box max and Q-box min (Table 1). Also the relative elasticity of parathyroids in comparison to unaffected surrounding thyroid tissue was lower than in case of BTL (Table 2). Parathyroids were usually much more elastic than thyroid parenchyma–mean ratio for Q-box mean was 0.3 and it was almost ten times higher for BTL. To conclude the issue, all elastographic parameters differed significantly between PAs and BTLs. Q-box mean seem to be most reliable due to inclusion of whole ROI for calculations–e.g., maximal measured value of Q-box mean for PAs was about twelve times higher than the median, in case of Qbox min–about five thousand times higher. There was no obvious advantage of using more sophisticated and time-consuming relative elasticities (lesion to adjacent thyroid tissue ratios).

**Table 1:** Comparison of selected sonographic paramethers between parathyroids and benign thyroid nodules

	Mean	SD	Median	*P*	Range
Q-box max [kPa]					
PA	12.9	16.1	8.0	<0.0001	0.0–76.5
BTL	39.3	46.1	28.0		0.0–260.0
Q-box mean [kPa]					
PA	5.2	7.2	2.1	<0.0001	0.0–25.6
BTL	24.3	33.8	13.3		0.0–193.9
Q-box min [kPa]					
PA	1.15	2.58	0.01	0.0002	0.0–13.5
BTL	11.11	20.50	1.73		0.0–115.0

PA parathyroid adenomas, *BTL* benign thyroid lesions, *SD* standard deviation

**Table 2:** Relative elasticity of parathyroid adenomas and benign thyroid lesions calculated as elasticity of the lesion divided by elasticity of neighbouring thyroid tissue

	Mean	SD	Median	*P*	Range
Q-box max [kPa]					
PA	0.48	0.61	0.26	0.0004	0.0–3.1
BTL	3.0	5.1	1.0		0.0–31.8
Q-box mean [kPa]					
PA	0.30	0.36	0.13	0.0001	0.0–1.3
BTL	2.8	3.9	0.78		0.0–13.5
Q-box min [kPa]					
PA	0.12	0.27	0.002	0.00003	0.0–1.0
BTL	3.7	7.8	0.24		0.0–34.9

*PA* parathyroid adenomas, *BTL* benign thyroid lesions, *SD* standard deviation

Elastographic pattern itself is not sufficient for reliable detection of PAs as many BTL also presents low stiffness. Taking into account that BTL are much more common than PAs, positive predictive value of SWE results close to average values for parathyroids would be low. However, in case of lesions presenting sonographic features typical for PAs or patients with clinical or laboratory signs typical for PH (e.g., nephrolithiasis, osteoporosis, hypercalcemia) high elasticity of the lesion should be considered as additional argument for further diagnostics. On the second hand, taking into account epidemiology of BTL and PAs, negative predictive value of low elasticity would be probably very high. Less than 5% of parathyroids presented stiffness exceeding the average Q-box mean value for BTLs so SWE can be valuable tool, which allows to exclude suspicion of PAs in most cases. None of 65 included enlarged parathyroids exceeded 30 kPa of Q-box mean value, what is rather average stiffness for BTL.

To our knowledge the current study is one of the few describing the use of elastography in the diagnosis of PA, and one out of two where Supersonic Imagine Aixplorer System has been evaluated. In the first study on the topic, written by Unluturk et al. [21], parathyroid adenomas were usually stiff lesions. To some extent these differences could be explained by different type of equipment used in this study. Strain elastography is more subjective and operator-dependent and the tissue stiffness is measured qualitatively–using the color scales, or as strain ratios. Finally, authors of this study did not compare pathologically enlarged parathyroids with benign thyroid lesions, but focused only in comparing above parameters in parathyroids enlarged due to different causes (adenoma, hyperplasia). In further studies [22–26] authors used a more objective modifications of the method–mainly based on Acoustic Radiation Force Technology (ARFI). Despite the elasticity was assessed with the use of shear waves–similarly as in the current study, there are many differences in compare to SWE; e.g., results are expressed as m/s in case of ARFI and in kPa in case of SWE, area of the ROI is small and limited in case of ARFI etc., so results obtained by both techniques can not be compared directly. According to Azizi et al. [22] parathyroid adenomas are less stiff than thyroid tissue (lower shear wave velocity), however there was no comparison with thyroid lesions. On the other hand, article published by Batur et al. [23] does not compare parathyroid adenomas with the thyroid tissue but only with the thyroid lesions. According to the authors parathyroid adenomas are stiffer than benign thyroid lesions but more elastic than thyroid cancer. In the study by Hapatoglou et al., the elasticity of parathyroid adenomas was significantly lower than thyroid tissue and similar to benign thyroid lesions [24]. On the other hand, Chandramohan et al. (using Virtual Touch tissue Imaging Quantitation-VITQ), presented higher elasticity of parathyroid adenomas in compare to the benign thyroid lesions [25]. Other studies were designed to estimate mean elasticity cut-off values of the parathyroid hyperplasia and adenomas in differentiating with benign thyroid nodules and reactive lymph nodes localized in the posterior part of the thyroid, with the promising results, but those studies were performed with the use of different technology to our study (Elastoscan Core Index and Virtual Touch tissue imaging quantification (VTIQ) method of SWE [26, 27].

As mentioned above, in compare to most of the studies, our assessment comprises comprehensive set of described parameters, comparison with the thyroid tissue as well as thyroid lesions, comparison of an absolute and relative elasticity between particular types of lesions. Also, the region-of-interest field dimensions are not fixed as in most of the ARFI devices (0.5 × 0.6 cm) and can be changed accordingly to the size of the analyzed lesion. The only study performed with the use of the same method and device (Supersonic Imagine Aixplorer system), on significantly smaller group of patients (*n* = 22), revealed similar results to our study–significantly higher parathyroid elasticity than surrounding thyroid tissue [28], but the authors did not include thyroid lesions in the assessment.

To conclude, SWE can be useful tool in the preliminary diagnostics of parathyroid adenomas. Enlarged parathyroids are significantly more elastic than benign thyroid lesions. Although the SWE itself does not allow for fully reliable differentiation of parathyroid adenomas and thyroid lesions and should be interpreted carefully, in context of other examinations, it can be considered as valuable, additional diagnostic tool. Particularly, low elasticity of the lesion constitutes feature with high negative prognostic value, allowing for reliable exclusion of suspicion of parathyroid adenomas. Further studies are strongly indicated due to discrepant results of particular studies, especially performed with different elastographic techniques.
